# The Relationship between Common Genetic Markers of Breast Cancer Risk and Chemotherapy-Induced Toxicity: A Case-Control Study

**DOI:** 10.1371/journal.pone.0158984

**Published:** 2016-07-08

**Authors:** Leila Dorling, Siddhartha Kar, Kyriaki Michailidou, Louise Hiller, Anne-Laure Vallier, Susan Ingle, Richard Hardy, Sarah J. Bowden, Janet A. Dunn, Chris Twelves, Christopher J. Poole, Carlos Caldas, Helena M. Earl, Paul D. P. Pharoah, Jean E. Abraham

**Affiliations:** 1 Centre for Cancer Genetic Epidemiology, University of Cambridge, Strangeways Research Laboratory, Cambridge, United Kingdom; 2 Cambridge Breast Unit and NIHR Cambridge Biomedical Research Centre, University of Cambridge NHS Foundation Hospitals, Cambridge, United Kingdom; 3 Cambridge Experimental Cancer Medicine Centre, Cambridge, United Kingdom; 4 Cancer Research UK Cambridge Research Institute, Li Ka Shing Centre, Robinson Way, Cambridge, United Kingdom; 5 Warwick Clinical Trials Unit, University of Warwick, Coventry, United Kingdom; 6 Cancer Research UK Clinical Trials Unit (CRCTU), University of Birmingham, Birmingham, United Kingdom; 7 Leeds Institute of Cancer and Pathology and Leeds Experimental Cancer Medical Centre, Leeds, United Kingdom; 8 Department of Electron Microscopy/Molecular Pathology, Cyprus Institute of Neurology and Genetics, Nicosia, Cyprus; Colorado State University, UNITED STATES

## Abstract

Ninety-four common genetic variants are confirmed to be associated with breast cancer. This study tested the hypothesis that breast cancer susceptibility variants may also be associated with chemotherapy-induced toxicity through shared mechanistic pathways such as DNA damage response, an association that, to our knowledge, has not been previously investigated. The study included breast cancer patients who received neoadjuvant/adjuvant chemotherapy from the Pharmacogenetic SNPs (PGSNPS) study. For each patient, a breast cancer polygenic risk score was created from the 94 breast cancer risk variants, all of which were genotyped or successfully imputed in PGSNPS. Logistic regression was performed to test the association with two clinically important toxicities: taxane- related neuropathy (n = 1279) and chemotherapy-induced neutropenia (n = 1676). This study was well powered (≥96%) to detect associations between polygenic risk score and chemotherapy toxicity. Patients with high breast cancer risk scores experienced less neutropenia compared to those with low risk scores (adjusted p-value = 0.06). Exploratory functional pathway analysis was performed and no functional pathways driving this trend were identified. Polygenic risk was not associated with taxane neuropathy (adjusted p-value = 0.48). These results suggest that breast cancer patients with high genetic risk of breast cancer, conferred by common variants, can safely receive standard chemotherapy without increased risk of taxane-related sensory neuropathy or chemotherapy-induced neutropenia and may experience less neutropenia. As neutropenia has previously been associated with improved survival and may reflect drug efficacy, these patients may be less likely to benefit from standard chemotherapy treatment.

## Introduction

Genome-wide association studies (GWAS) provide an empirical approach for identifying moderate risk alleles for a variety of widespread complex diseases and traits. Meta-analyses of 11 breast cancer GWAS (15,748 cases and 18,084 controls) and 41 studies in the Breast Cancer Association Consortium (BCAC) (46,785 cases and 42,892 controls) have confirmed 94 breast cancer susceptibility loci (p-value<5 x 10^−8^)[1–3]. Effect sizes of each genetic locus are generally modest (OR≤1.34), but together they explain approximately 16% of the excess familial risk of breast cancer. In a recent study by Mavaddat *et al*, a breast cancer polygenic risk score was created using 77 breast cancer risk variants. Women in the highest 1% of the risk score were 3.6 times more likely to develop breast cancer than women in the middle quintile [4].

We have hypothesised that genetic determinants of breast cancer incidence may be associated with the risk of chemotherapy-induced toxicity. This was based on the concept that variation in genes involved in pathways such as the repair of DNA damage may be important in both the mechanisms of tumour formation and proliferation and in the response to DNA damage induced by chemotherapy. For example, cyclophosphamide is an alkylating agent used to treat a variety of cancers including breast cancer. Cyclophosphamide interferes with DNA replication by forming intra-strand and inter-strand DNA cross-links, preventing tumour proliferation. The DNA cross-link repair 1B (*DCLRE1B*) gene is involved in repair of inter-strand cross-links and a common allele of this gene (rs11552449) is associated with increased risk of breast cancer [1]. The precise functional effect of this variant is unknown but patients with this mutation may also be less able to repair inter-strand cross-links induced in normal tissue by cyclophosphamide during treatment for breast cancer, resulting in increased toxicity.

Further, specific mutations that influence the risk of breast cancer developing may also affect genes in specific drug metabolism pathways. For example, it is known that polyadenosine 5’diphosphoribose polymerisation (PARP) enzymes play an important role in the repair of single strand breaks. PARP inhibitors (PARPi) target DNA homologous repair pathways, by preventing repair of single strand breaks leading to problems downstream with double strand repair. PARPi work, therefore, in synergy with DNA damaging agents like platinums, which also cause strand breaks. In patients with rare *BRCA1* or *BRCA2* mutations, the already compromised homologous repair pathways allow PARPi to work particularly effectively leading to “synthetic lethality” [5]. Thus, such genetic mutations both increase susceptibility to breast cancer and enable a better response to certain treatments, although the drug toxicity profile of patients carrying these mutations is, as yet, unclear. Variants of genes that play a role in drug metabolism may also lie in pathways unrelated to DNA repair.

To date, chemotherapy toxicity GWAS have had limited success in identifying common genetic variants that significantly influence a patient’s risk of toxicity [6–11]. This is mainly due to lack of statistical power stemming from small samples and the requirement for stringent p-value thresholds for obtaining statistical significance. No single nucleotide polymorphisms (SNPs) reaching genome-wide significance have been independently replicated in validation samples to accepted GWAS levels of significance.

The aim of this study was to look for associations between common genetic variants known to increase the risk of breast cancer and chemotherapy-induced toxicity using patient samples from the Pharmacogenetic SNPs (PGSNPS) study, one of the largest chemotherapy toxicity GWAS to date. Common genetic variants have small individual effects on breast cancer so it is likely that they will also have small individual effects on chemotherapy toxicity. Thus, to increase the power to detect an association between genotype and toxicity, variants were combined in a polygenic risk score. Whilst for most chemotherapeutic agents there is a presumed mechanism of action, it is widely accepted that we do not have a complete understanding of all the mechanisms by which the majority of the agents function. Further, the precise impact of many of the breast cancer variants on the gene in which they lie and the mechanisms underlying the individual associations between each variant and breast cancer risk are as yet unknown. Thus, all breast cancer risk variants were included in our analyses.

## Materials and Methods

### Patients

The study cohort consisted of female breast cancer patients from PGSNPS, a large study that was set up to investigate the role of germline variants in chemotherapy toxicity [12]. The PGSNPS sample includes 2354 female patients from four UK breast cancer chemotherapy trials: NEAT [13], BR9601 [13], tAnGo [14] and Neo-tAnGo [15]. S1 Table and S1 Fig (see Supporting Information) summarise PGSNPS and the clinical trial regimens. In brief, patients in NEAT and BR9601 received either six or eight cycles of cyclophosphamide, methotrexate and 5-fluouracil (CMF) or four cycles of epirubicin (E) followed by four cycles of CMF, while patients in tAnGo and Neo-tAnGo received either four cycles of EC followed by four cycles of paclitaxel (T) with or without gemcitabine (±G) or four cycles of T±G followed by four cycles of T. DNA samples were collected along with demographic, tumour and treatment information, chemotherapy toxicity scores and relapse and survival times. An additional 56 patients who were not taking part in a clinical trial were recruited from the Cambridge University Hospitals NHS Foundation Trust breast unit using the same clinical response forms. These patients received four cycles of epirubicin (E) followed by four cycles of cyclophosphamide, methotrexate and 5-fluorouracil (CMF).

### Ethics and data availability

PGSNPS [Pharmacogenetics of Early Breast Cancer Chemotherapy–reference number 05/Q0104/1] was approved by the NRES Committee East of England—Cambridge East. All participants provided informed consent to take part in PGSNPS.

The data used in this study is held by the Trial Management Group for PGSNPS, where the original concept for this analysis was designed. Any access requires appropriate ethical approvals and would be assessed by the Trial Management Group which includes the respective Chief Investigators of the clinical trials and PGSNPS. Transfer of data would require a specific Data Transfer Agreement.

### Toxicity phenotypes

This study investigated two common and clinically important chemotherapy-induced toxicities: neutropenia and taxane-related sensory neuropathy (for the purposes of this study, this will be referred to as simply “neuropathy” from now on). For all patients, toxicity information was collected prospectively and graded using the National Cancer Institute Common Toxicity Criteria for Adverse Events (NCI CTCAE) version 2 or 3, depending on the clinical trial from which the patient was recruited into PGSNPS. Rates and grades of neutropenia were recorded in 1676 patients who received any of the trial chemotherapy regimens in NEAT, tAnGo and Neo-tAnGo (data for neutropenia was not available from BR9601) or were not in a trial and received E-CMF. Rates and grades of neuropathy were recorded in 1279 patients who received a paclitaxel-containing regimen (tAnGo and Neo-tAnGo).

### Genotyping, quality control and imputation

Samples were genotyped using the Affymetrix 6.0 SNP array. Quality control procedures were applied to remove variants that were missing in >5% of samples; had minor allele frequency (MAF) < 1%; or had MAF<5% and were missing in >1% samples. Variants were also removed if their genotype frequencies deviated from those expected under Hardy-Weinberg equilibrium (p-value < 10^−5^). Samples were removed that had >10% of all variants missing. Principle components analysis (PCA) was used to identify and exclude individuals with non-European ancestry and control for population substructure. Genome coverage was increased by imputation using SHAPEIT [16] and IMPUTE v2 [17] with the 1000 Genomes reference panel [18]. Genotype dosages of the breast cancer risk alleles were extracted from the imputed data.

### Statistical methods

To quantify each patient’s genetic risk of breast cancer, polygenic risk scores were created by summing the patient’s risk allele dosages across all the variants. Two risk scores were calculated:

1. Non-weighted: ${risk\;score}_{i}=\sum_{1}^{j}G_{i}$
2. Weighted: ${weighted\;risk\;score}_{i}=\sum_{1}^{j}\beta_{j}G_{i}$

for patient *i*,

where *j* = variant 1..94

*β_j_* = the per-allele log-odds ratio for risk of breast cancer associated with variant *j*

*G* = risk allele dosage

The log-odds ratios used to weight the risk score were taken directly from the report by Mavaddat *et al* [4] who tested the association of each variant with breast cancer risk while adjusting for the effect of other variants (see Table 1). Seventeen variants have been identified since Mavaddat *et al* performed their analysis [2,3]. For these, the log-odds ratios used were those reported by Michailidou et al [3].

**Table 1:** Genetic variants known to influence risk of breast cancer.

Variant	Nearestgene	Chr	Position (build 37)	Breast cancer risk allele	Published odds ratio^a^	PGSNPS risk allele frequency	PGSNPS imputation r^2^ ^c^
rs616488	*PEX14*	1	10566215	A	1.06	0.67	0.96
rs11552449	*PTPN22-BCL2L15-AP4B1-DCLRE1B-HIPK1*	1	114448389	T	1.08	0.18	0.94
rs11249433	None	1	121280613	G	1.1	0.43	0.81
rs12405132	*RNF115*	1	145644984	C	1.05^b^	0.63	1
rs12048493	*OTUD7B*	1	149927034	C	1.07^b^	0.34	0.52
rs6678914	*LGR6*	1	202187176	G	1.01	0.40	0.99
rs4245739	*MDM4*	1	204518842	C	1.03	0.28	1
rs72755295	*EXO1*	1	242034263	G	1.15^b^	0.04	0.72
rs12710696	*OSR1*	2	19320803	A	1.04	0.37	1
rs4849887	*INHBB*	2	121245122	C	1.09	0.92	1
rs2016394	*METAP1D-DLX1-DLX2*	2	172972971	G	1.05	0.55	0.87
rs1550623	*CDCA7*	2	174212894	A	1.06	0.85	1
rs1045485	*CASP8*^d^	2	202149589	G	1.04	0.87	0.99
rs13387042	IGFBP5^d^	2	217905832	A	1.14	0.51	1
rs16857609	*DIRC3*	2	218296508	T	1.07	0.28	0.99
rs6762644	*ITPR1-EGOT*	3	4742276	G	1.07	0.41	0.99
rs4973768	*SLC4A7*	3	27416013	T	1.09	0.49	0.99
rs12493607	*TGFBR2*	3	30682939	C	1.05	0.34	0.99
rs6796502	*PRSS42*	3	46866866	G	1.09^b^	0.91	0.96
rs1053338	*ATXN7*	3	63967900	G	1.08^b^	0.15	0.99
rs9790517	*TET2*	4	106084778	T	1.05	0.20	0.99
rs6828523	*ADAM29*	4	175846426	C	1.1	0.89	1
rs10069690	*TERT*^d^	5	1279790	T	1.02	0.25	0.66
rs7726159	*TERT*^d^	5	1282319	A	1.04	0.36	0.75
rs2736108	*TERT*^d^	5	1297488	C	1.07	0.73	0.78
rs13162653	*MARCH11*	5	16187528	G	1.05^b^	0.57	0.97
rs2012709	*SUB1*	5	32567732	T	1.05^b^	0.49	0.99
rs10941679	None	5	44706498	G	1.12	0.26	0.95
rs889312	*MAP3K1*^d^	5	56031884	C	1.12	0.30	0.99
rs10472076	*RAB3C*	5	58184061	C	1.04	0.38	0.94
rs1353747	*PDE4D*	5	58337481	T	1.09	0.89	0.99
rs7707921	*ATG10*	5	81538046	A	1.08^b^	0.75	0.99
rs1432679	*EBF1*	5	158244083	G	1.07	0.44	0.99
rs11242675	*FOXQ1*	6	1318878	T	1.06	0.63	0.99
rs204247	*RANBP9*	6	13722523	G	1.05	0.45	1
rs9257408	None	6	28926220	C	1.05^b^	0.37	0.98
rs17529111	None	6	82128386	G	1.05	0.22	0.98
rs12662670	*ESR1*^e^	6	151918856	G	1.14	0.09	0.99
rs2046210	*ESR1*^e^	6	151948366	A	1.05	0.38	1
rs6964587	*AKAP9*	7	91630620	T	1.05^b^	0.40	1
rs4593472	*LINC-PINT*	7	130667121	C	1.05^b^	0.64	1
rs720475	*ARHGEF5-NOBOX*	7	144074929	G	1.06	0.74	1
rs9693444	None	8	29509616	A	1.07	0.35	0.99
rs13365225	*KCNU1*	8	36858483	A	1.05^b^	0.84	1
rs6472903	*CASC9*	8	76230301	T	1.1	0.84	0.93
rs2943559	*HNF4G*	8	76417937	G	1.13	0.09	1
rs13267382	None	8	117209548	A	1.05^b^	0.36	0.93
rs13281615	*MYC*^d^	8	128355618	G	1.1	0.43	0.99
rs11780156	*MYC*^d^	8	129194641	T	1.07	0.19	1
rs1011970	*CDKN2A/B*	9	22062134	T	1.05	0.18	0.99
rs10759243	*KLF4*^d^	9	110306115	A	1.05	0.29	1
rs865686	*KLF4*^d^	9	110888478	T	1.11	0.64	0.99
rs2380205	*ANKRD16*	10	5886734	C	1.02	0.57	1
rs7072776	*MLLT10-DNAJC1*	10	22032942	A	1.06	0.30	1
rs11814448	*DNAJC1*	10	22315843	C	1.22	0.02	0.98
rs10995190	*NRBF2*^d^	10	64278682	G	1.17	0.87	0.99
rs704010	*ZMIZ1*	10	80841148	T	1.07	0.42	1
rs7904519	*TCF7L2*	10	114773927	G	1.06	0.48	0.99
rs11199914	*FGFR2*^d^	10	123093901	C	1.06	0.70	0.99
rs2981579	*FGFR2*^d^	10	123337335	A	1.25	0.44	0.99
rs3817198	*LSP1*	11	1909006	C	1.07	0.34	1
rs3903072	*DKFZp761e198-OVOLI-SNX32-CFL1-MUS81*	11	65583066	G	1.06	0.56	1
rs78540526	*CCND1*^d^	11	69331418	T	1.18	0.08	0.98
rs554219	*CCND1*^d^	11	69331642	G	1.12	0.13	0.99
rs75915166	*CCND1*^d^	11	69379161	A	1.024	0.07	0.95
rs11820646	*BARX2*	11	129461171	C	1.05	0.60	0.99
rs12422552	None	12	14413931	C	1.03	0.29	0.91
rs10771399	*PTHLH*	12	28155080	A	1.16	0.89	0.99
rs17356907	*NTN4*	12	96027759	A	1.1	0.72	1
rs1292011	None	12	115836522	A	1.08	0.58	1
rs11571833	*BRCA2-N4BP2LI-N4BP2L2*	13	32972626	T	1.26	0.01	0.99
rs2236007	*PAX9-SLO25A21*	14	37132769	G	1.09	0.81	0.98
rs2588809	*RAD51L1*	14	68660428	T	1.07	0.17	1
rs999737	*RAD51L1*	14	69034682	C	1.08	0.76	0.99
rs941764	*CCDC88C*	14	91841069	G	1.06	0.35	0.99
rs11627032	*RIN3*	14	93104072	T	1.06^b^	0.76	0.99
rs3803662	*TOX3*	16	52586341	A	1.23	0.30	1
rs17817449	*MIRI972-2-FTO*	16	53813367	T	1.08	0.61	0.99
rs11075995	*FTO*	16	53855291	T	1.04	0.24	0.99
rs13329835	*CDYL2*	16	80650805	G	1.08	0.23	0.99
rs146699004	*TEFM*	17	29230520	GGT	1.08^b^	0.81	0.87
rs6504950	*COX11*^e^	17	53056471	G	1.07	0.72	1
rs745570	*CBX8*	17	77781725	A	1.05^b^	0.50	1
rs527616	None	18	24337424	G	1.04	0.66	0.94
rs1436904	*CHST9*	18	24570667	T	1.06	0.60	1
rs6507583	*SETBP1*	18	42399590	A	1.10^b^	0.93	0.99
rs8170	*ABHD8/ANKLE1*^e^	19	17389704	A	1.03	0.20	0.99
rs2363956	*ABHD8/ANKLE1*^e^	19	17394124	T	1.03	0.51	0.96
rs4808801	*SSBP4-ISYNA1-ELL*	19	18571141	A	1.07	0.66	1
rs3760982	*C19orf61-KCNN4-LYPD5-ZNF283*	19	44286513	A	1.06	0.49	0.99
rs2823093	*NRIP1*	21	16520832	G	1.08	0.75	0.97
rs17879961	*CHEK2*	22	29121087	G	1.36	0.001	0.86
rs132390	*EMID1-RHBDD3-EWSR1*	22	29621477	C	1.11	0.04	0.78
rs6001930	*MKL1*	22	40876234	C	1.13	0.10	1

^a^Adjusted breast cancer odds ratios from Mavaddat *et al* (4)

^b^Unadjusted odds ratios from BCAC meta-analysis (1–3)

^c^Mean imputation r^2^ from IMPUTE2 (r^2^ = 1 for genotyped SNPs)

^d^published target gene

^e^known target gene, not yet published

*Chr*: *chromosome*.

Neutropenia and neuropathy grades were dichotomised into cases (neutropenia grade ≥3, neuropathy grade ≥2) and controls (neutropenia grades 0–2, neuropathy grades 0–1) (see Table 2). Logistic regression was used to test the association between polygenic risk score and toxicity case status for neutropenia and neuropathy, respectively. Each of the 94 genetic variants was also tested separately for association with neutropenia and neuropathy. For multivariable analysis, pre-specified important non-genetic covariates were included in the models. The neutropenia analysis was adjusted for trial and patient age and the neuropathy analysis was adjusted for trial, pre-treatment body mass index (BMI) and the first two principle components to control for population substructure. Per-allele odds ratios (ORs) and 95% confidence intervals (CIs) are presented for the non-weighted polygenic risk score and individual variants. ORs and CIs corresponding to a one standard deviation (SD) increase in risk score are presented for the weighted polygenic risk score.

**Table 2:** Distribution of chemotherapy-induced neutropenia and taxane-related sensory neuropathy in the PGSNPS sample according to the National Cancer Institute Common Toxicity Criteria for Adverse Events (NCI CTCAE) version 2/3.

NCI CTCAE grade	Neutropenia; total N = 1676 n (%)	Neuropathy; total N = 1279 n (%)
0	733 (43.7)	271 (21.2)
1	199 (11.9)	648 (50.7)
2	245 (14.6)	304 (23.7)
3	293 (17.5)	56 (4.4)
4	206 (12.3)	0 (0)
Toxicity cases (moderate-severe toxicity)	grade ≥3	grade ≥2
	499 (29.8)	360 (28.1)

#### Pathway analysis

Interesting associations between polygenic risk and toxicity were followed up with exploratory pathway analysis to investigate whether a subset of the breast cancer variants, lying in a common pathway, were responsible for the observed association. The breast cancer variants were mapped to the genes in which they lay or to the nearest gene if they were intergenic. These variant-gene pairs were ranked using the p-value for association between each variant and toxicity, from most strongly to least strongly associated, regardless of the direction of effect on toxicity. Where more than one variant mapped to the same gene, the most significant toxicity-associated variant was used for ranking. The top 50% of the ranked genes were entered into the Database for Annotation, Visualization and Integrated Discovery (DAVID) version 6.7 functional annotation tool [19,20]. DAVID draws functional annotations from various online databases to group genes that are in the same biological pathway and performs a Fisher’s exact test to determine whether genes from any particular pathway are overrepresented in the user’s list of genes. A Fisher exact p-value≤0.05 identified pathways that were significantly enriched among the top genes for toxicity.

#### Statistical Power

This study was well powered to detect significant associations between breast cancer polygenic risk score and the toxicity endpoints examined. Assuming a 30% prevalence of moderate-severe toxicity (neutropenia ≥3, neuropathy grade ≥2) in breast cancer patients, the power to detect a small difference of 0.1 in mean risk score between patients with moderate-severe toxicity and patients with no or mild toxicity, at p-value<0.05, would be 96% in the neuropathy sample and 99% in the neutropenia sample. This difference in mean risk score is equivalent to a relative risk of moderate-severe toxicity of 1.1 for patients with a higher polygenic risk score.

## Results

**Fig 1:**
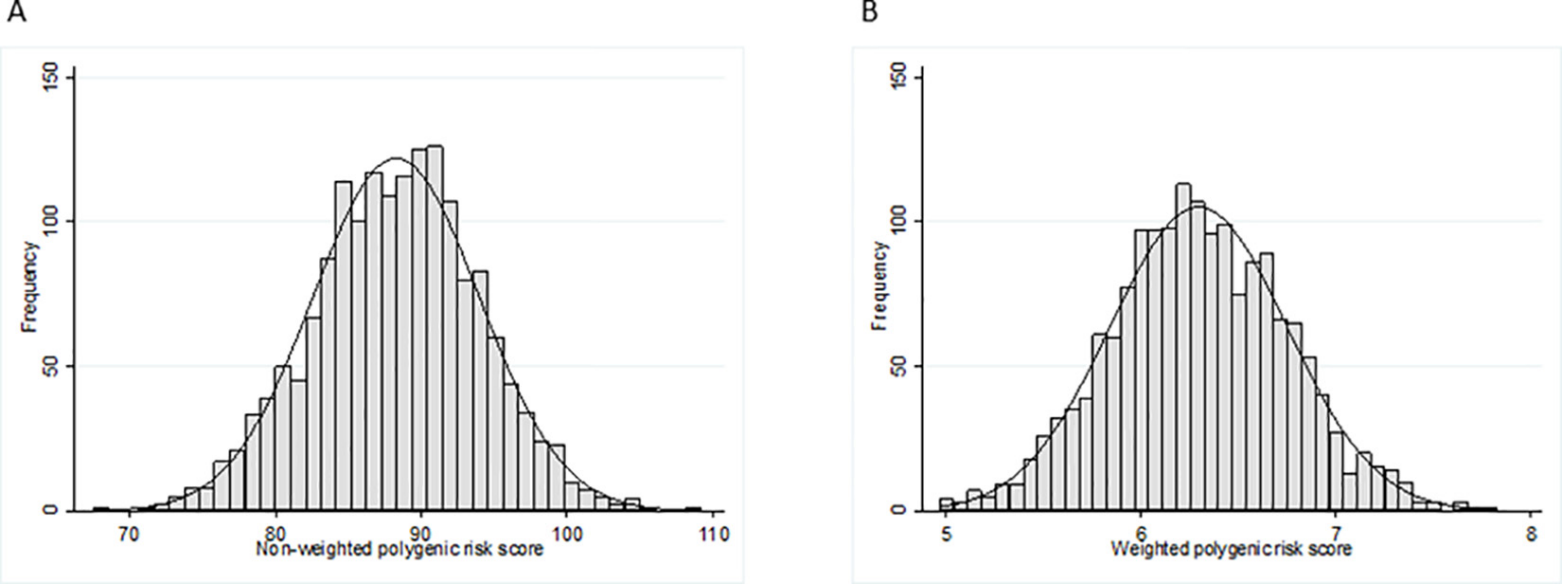
Distribution of polygenic risk scores in the PGSNPS cohort. A) Non-weighted polygenic risk score. B) Weighted polygenic risk score.

The total number of patients included in this study was 1677. Patient characteristics are summarised in S2 Table (see Supporting Information). All 94 genetic variants known to increase the risk of breast cancer were genotyped or successfully imputed (IMPUTE2 info metric>0.5) in the PGSNPS sample. The variants and information about MAF and imputation certainty can be found in Table 1. Fig 1 shows the approximately normal distribution of the two polygenic risk scores in the PGSNPS patients.

The non-weighted risk score was significantly associated with a decreased risk of neutropenia (grade ≥3) on univariable analysis (per-allele OR = 0.98; 95% CI = (0.96, 0.99); p = 0.04) (Table 3). This finding was not nominally significant when adjusted for age and trial (p = 0.06) but the effect size was the same. The weighted risk score was not significantly associated with neutropenia (grade ≥3) but the effect was in the same direction as with the non-weighted score. Neither the non-weighted nor the weighted risk score was associated with neuropathy (OR = 0.99 (0.97, 1.01); p = 0.48 and OR = 0.99 (0.95, 1.02); p = 0.47, respectively). None of the individual genetic variants were significantly associated with neuropathy or neutropenia at the p<5 x 10^−4^ level.

**Table 3:** Association of polygenic risk scores with chemotherapy-related neutropenia and taxane-related sensory neuropathy.

	Odds ratio (95% confidence interval) & p-value^a^			
	Non-weighted risk score		Weighted risk score	
	unadjusted	adjusted^b^	unadjusted	adjusted^b^
neutropenia (n = 1676)	0.98 (0.96, 0.99) p = 0.04	0.98 (0.96, 1.00) p = 0.06	0.98 (0.95, 1.01) p = 0.16	0.98 (0.95, 1.01) p = 0.20
neuropathy (n = 1279)	0.99 (0.97, 1.01) p = 0.41	0.99 (0.97, 1.01) p = 0.48	0.98 (0.95, 1.02) p = 0.37	0.99 (0.95, 1.02) p = 0.47

^a^Non-weighted risk score: per-allele odds ratio and confidence interval; Weighted risk score: per-standard deviation odds ratio and confidence interval

^b^Neutropenia: adjusted for age and trial; neuropathy: adjusted for body mass index, trial and first two principle components.

### Pathway analysis

Pathway analysis was performed to investigate whether a specific molecular pathway was driving the trend between increased breast cancer risk and reduced risk of neutropenia. The individual breast cancer risk variants were ranked according to their p-value for association with neutropenia and the ranked variant-gene pairs were compiled to create a list of 76 genes. Table 4 shows the top 50% (n = 38) of genes in the ranked list. The DAVID functional overrepresentation tool [19,20] was used to annotate the top 38 genes and identified the p53 signalling pathway as the most strongly enriched pathway (Fisher exact p-value = 0.004). Three genes (*CCND1*, *CHEK2*, *MDM4*) in the p53 signalling pathway appeared in the top 38 genes. However, this observed enrichment did not remain significant following Bonferroni correction for the multiple pathways tested by the DAVID tool (corrected p53 signalling pathway p-value = 0.13).

**Table 4:** Top 38 genes according to p-value for association between highest-ranking breast cancer risk variant and neutropenia.

Gene	Breast cancer risk variant	Variant association with neutropenia	
		Odds Ratio	P-value
*ZMIZ1*	rs704010	1.20	0.02
*KCNN4*	rs3760982	0.85	0.03
*MAP3K1*	rs889312	1.19	0.04
*DCLRE1B*	rs11552449	0.81	0.05
*KCNU1*	rs13365225	0.81	0.05
*DLX2*	rs2016394	0.86	0.06
*ITPR1*	rs6762644	1.15	0.08
*MYC*	rs13281615	1.14	0.10
*RANBP9*	rs204247	1.13	0.10
*CASC9*^**a**^	rs6472903	0.84	0.10
*CBX8*	rs745570	0.89	0.13
*SNX32*	rs3903072	0.90	0.17
*SLC4A7*	rs4973768	1.11	0.18
*TET2*	rs9790517	0.89	0.22
*KLF4*	rs10759243	1.10	0.23
*TERT*	rs2736108	0.89	0.26
*ANKRD16*	rs2380205	0.92	0.27
*CDYL2*	rs13329835	0.90	0.27
*NRIP1*	rs2823093	0.91	0.29
*CHST9*	rs1436904	0.93	0.34
***CCND1***	**rs78540526**	**1.14**	**0.37**
*FGFR2*	rs2981579	1.07	0.37
*DNAJC1*	rs11814448	0.74	0.37
*HNF4G*	rs2943559	0.88	0.38
*ATXN7*	rs1053338	0.91	0.38
*AKAP9*	rs6964587	1.07	0.38
*MKL1*	rs6001930	0.90	0.41
*LGR6*	rs6678914	1.06	0.45
*FOXQ1*	rs11242675	0.94	0.46
*ANKLE1*	rs8170	1.07	0.50
*RNF115*	rs12405132	0.95	0.51
*SETBP1*	rs6507583	1.10	0.52
*ADAM29*	rs6828523	0.92	0.52
*NRBF2*	rs10995190	0.93	0.53
*ESR1*	rs12662670	0.92	0.53
***CHEK2***	**rs17879961^**b**^**	**1.36x10^-19^**	**0.54**
*PTHLH*	rs10771399	0.93	0.56
*IGFBP5*	rs13387042	0.96	0.59
***MDM4***	**rs4245739**	**0.96**	**0.60**

^a^CASC9 not mapped by DAVID tool so excluded from pathway analysis

^b^variant frequency in PGSNPS = 0.001

*p53 signalling pathway genes highlighted in bold*.

## Discussion

**Fig 2:**
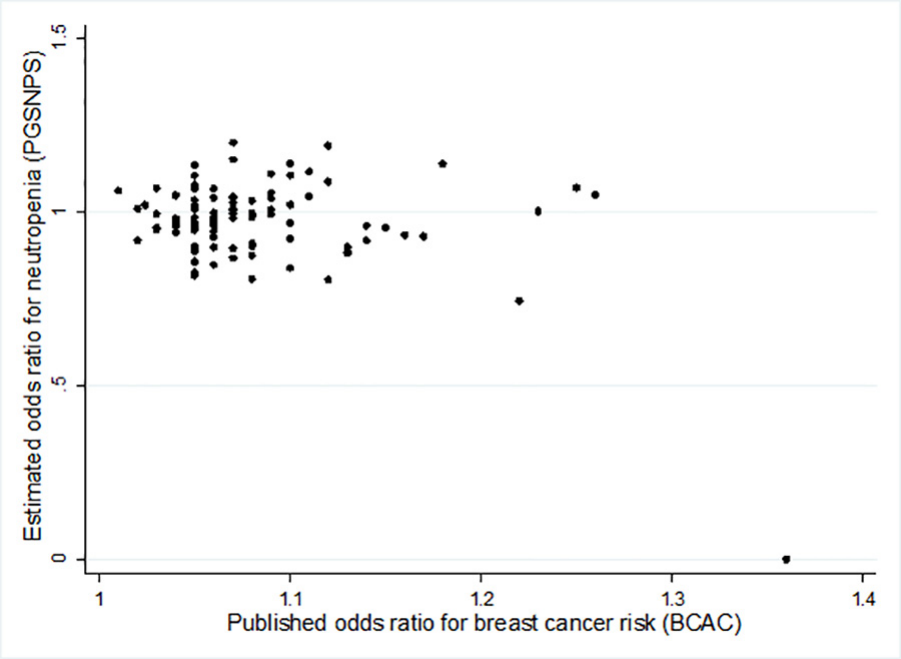
Scatter plot comparing variant-associated odds ratios for breast cancer risk, published by the Breast Cancer Association Consortium (BCAC), with odds ratios for neutropenia risk estimated in the Pharmacogenetic SNPs (PGSNPS) study.

The hypothesis behind this study was that common genetic variants known to increase the risk of breast cancer may also increase the likelihood of developing treatment-related toxicity following chemotherapy for breast cancer. In this well powered study, no evidence was found for an association between common variants known to increase breast cancer risk and taxane-related sensory neuropathy in the PGSNPS cohort. Interestingly, and contrary to our hypothesis, there was some evidence of a relationship between carrying an increased number of breast cancer risk alleles and decreased risk of experiencing chemotherapy-induced neutropenia grade ≥3 (OR = 0.98; 95% CI = (0.96, 1.00)). Weighting the alleles by the estimate of their effect on breast cancer risk reduced the strength of this association. This suggests that the magnitude of effect that these variants have on risk of neutropenia is not equal to their magnitude of effect on risk of breast cancer. This is demonstrated in Fig 2, which shows the effects that the individual variants have on breast cancer risk (as reported by Mavaddat *et al* 2015) plotted against their effects on neutropenia in the PGSNPS sample; there is no visible relationship between the effects. Pathway analysis did not identify any significant pathway enrichment in the genes representing the top-ranked breast cancer risk variants.

None of the individual variants were significantly associated with chemotherapy-induced toxicity. The original work that confirmed the association of the 94 genetic variants with breast cancer risk was performed by BCAC and based on samples of over 100,000 patients. In contrast, the PGSNPS breast cohort studied in the current analysis has fewer than 2,000 patients. Therefore, the power to detect a true association at genome-wide significance is much lower than that of the breast cancer susceptibility studies. With an increased sample size, there would be greater power to detect strong associations between individual variants and chemotherapy toxicity.

The observed association between polygenic breast cancer risk and decreased neutropenia suggests that breast cancer patients who present with a high genetic risk of breast cancer, conferred by common variants, can safely receive standard chemotherapy and may experience less neutropenia compared to patients with low genetic risk of breast cancer. There is strong evidence to support the relationship between neutropenia or leukopenia and improved survival [21–23]. Abraham *et al* have shown that in a cohort of over 6000 early breast cancer patients from randomised clinical trials, those who achieved neutropenia grade ≥3 during their treatment had statistically significant improved relapse-free survival (hazard ratio = 0·86; 95% CI = (0·76–0·97); p = 0·02) [23]. In the current study population, expanded clinical and survival data was available for 1450 patients. After adjusting for non-genetic predictors of survival, the 29% of breast cancer patients who experienced neutropenia grade ≥3 had longer relapse-free survival compared to the 71% who did not experience neutropenia grade ≥3 (HR = 0.71; 95% CI = (0.54–0.94); p = 0.02). Neutropenia may therefore be a surrogate marker of efficacy, although the mechanisms underlying the association between neutropenia and survival are unclear. The hypothesis that neutropenia may reflect efficacy is supported by a recent prospective randomised phase III trial of tailored and dose-dense versus standard tri-weekly adjuvant chemotherapy for high risk breast cancer. In the tailored and dose-dense therapy arm of the trial, where a patient had a toxicity of grade 2 or less, the chemotherapy dose was escalated. The results of the trial showed that the tailored approach resulted in an improvement in all studied efficacy endpoints [24].

**Fig 3:**
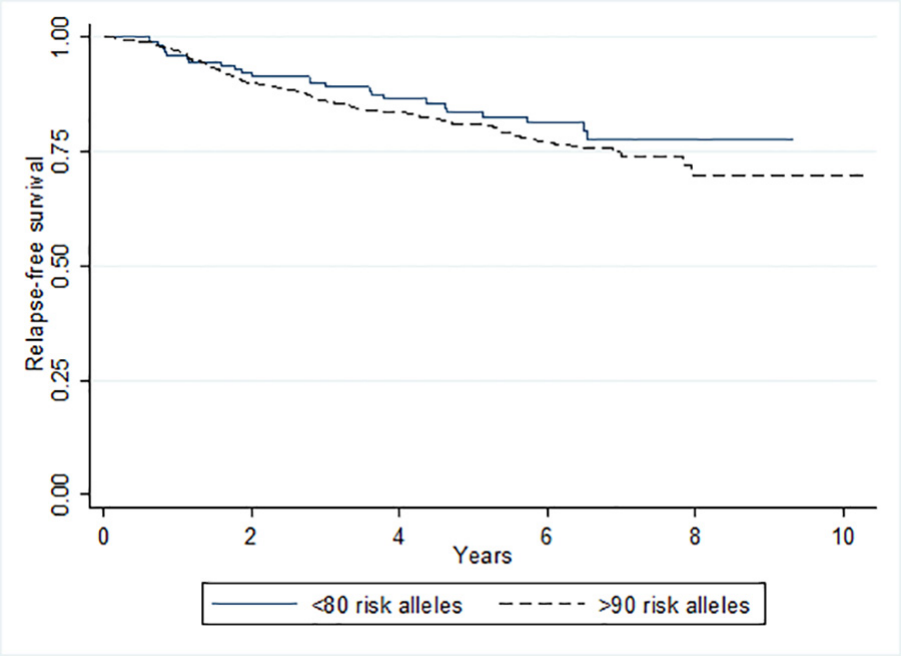
Kaplan-Meier plot comparing relapse-free survival in patients carrying >90 risk alleles to those carrying <80 risk alleles.

Given the potential relationship between neutropenia and clinical outcome, the finding that patients with high polygenic risk of breast cancer experience less neutropenia may, firstly, reflect the fact that for some patients standard chemotherapy regimens are sub-optimal and, secondly, suggests that genetic risk of cancer may potentially distinguish these patients, who may tolerate more intense chemotherapy that could improve survival. If this is the case, common breast cancer risk variants may be a useful tool for predicting which patients are likely to have poorer prognosis. We evaluated the relationship between the breast cancer risk polygenic score and relapse-free survival in the same cohort of patients. The polygenic risk score was predictive of relapse-free survival such that patients who have an increased risk of breast cancer (and therefore lower risk of neutropenia) tended to have shorter relapse-free survival (HR = 1.02; 95% CI = (1.00–1.04); p = 0.06). This equates to a 23% increase in risk of relapse or death for every 10 extra risk alleles that a patient carries (HR = 1.23; 95% CI = (0.99–1.51); p = 0.06). This difference in hazards is illustrated in a Kaplan-Meier plot in Fig 3. After adjusting for neutropenia case-control status, this relationship was weakened slightly; patients carrying an extra 10 risk alleles had 21% increase in risk of relapse or death (HR = 1.216; 95% CI = (0.98–1.49); p = 0.08). These results support the hypothesis that neutropenia is a marker of efficacy of chemotherapy and that efficacy could be predicted by breast cancer polygenic risk. However, a large study with more power to detect subtle survival effects is required to confirm these results.

In conclusion, for breast cancer patients who are carrying common genetic variants known to increase the risk of breast cancer, standard chemotherapy for breast cancer, although safe, may not be adequately effective. It is likely that there are less common variants and rare mutations that have large effects on toxicity response to chemotherapy and these may prove more useful for predicting patient drug response in the clinic. Thus, targeted sequencing of candidate genes or whole-exome/genome sequencing in large patient samples should be a next step in the search for pharmacogenetic determinants of chemotherapy toxicity.

## Supporting Information

S1 Fig Summary of PGSNPS clinical trials regimens. (DOCX)

S1 Table Summary of Clinical Trials contributing to PGSNPS. (DOCX)

S2 Table Patient Characteristics. (DOCX)
