# Supplementary material for: The Relationship between Common Genetic Markers of Breast Cancer Risk and Chemotherapy-Induced Toxicity: A Case-Control Study
Source: PLoS One. 2016 Jul 8;11(7):e0158984. doi: 10.1371/journal.pone.0158984 (PMC4938564; doi:10.1371/journal.pone.0158984)
Supplement: S1 Fig — (DOCX) [file pone.0158984.s001.docx]

**S1 Fig: Summary of PGSNPS clinical trial regimens**

**NEAT**

**BR9601**

**tAnGo**

**Neo-tAnGo**

Eligibility Criteria

1. Early stage breast cancer
2. Tumour completely excised
3. Certain indication for chemotherapy

SURGERY

Stratification

1. Nodal status: negative; 1-3; 4+
2. Age: ≤50 years; >50 years
3. Radiotherapy timing: none; concurrent; sequential; to be randomised into a radiotherapy sequencing trial
4. Randomising centre

Randomisation

**Arm A**

**6 x classic CMF**

C: 600mg/m^2^ day 1 & 8 q3 weekly OR 100mg/m^2^ orally every day for 14 days

M: 40mg/m^2^ day 1 q3 weekly

F: 600mg/m^2^ day 1 & 8 q3 weekly

**Arm B**

**4 x E followed by 4 x CMF**

E: 100mg/m^2^ q3 weekly

CMF: as per Arm A

Eligibility Criteria

1. Early stage breast cancer
2. Tumour completely excised
3. Certain indication for chemotherapy

SURGERY

Stratification

1. Nodal status: negative; 1-3; 4+
2. Age: ≤50 years; >50 years
3. Radiotherapy timing: none; concurrent; sequential; to be randomised into a radiotherapy sequencing trial
4. Randomising centre

Randomisation

**Arm A**

**8 x modified CMF**

C: 750mg/m^2^ day 1 q3 weekly

M: 50mg/m^2^ day 1 q3 weekly

F: 600mg/m^2^ day 1 q3 weekly

**Arm B**

**4 x E followed by 4 x modified CMF**

E: 100mg/m^2^ q3 weekly

CMF: as per Arm A

Eligibility Criteria

1. Early stage breast cancer
2. Tumour completely excised
3. Certain indication for chemotherapy

SURGERY

Stratification

1. Country of randomising hospital: England; Scotland; Wales; Northern Ireland; Republic of Ireland
2. Nodal status: negative; 1-3; 4+
3. Age: ≤50 years; >50 years
4. ER status: negative; weakly positive; positive
5. HER2 status: +++; other; unmeasured
6. Radiotherapy: yes; no

**Arm A**

**4 x EC followed by 4 x T**

E: 90mg/m^2^ q3 weekly

C: 600mg/m^2^ q3 weekly

T: 175mg/m^2^ q3 weekly

**Arm B**

**4 x EC followed by 4 x T+G**

E: 90mg/m^2^ q3 weekly

C: 600mg/m^2^ q3 weekly

T: 175mg/m^2^ q3 weekly

G: 1250mg/m^2^ day 1 & 8 q3 weekly

Randomisation

Eligibility Criteria

1. Histologically confirmed invasive breast cancer
2. T2 tumour and above
3. Diameter >20mm

Stratification

1. Age: ≤50 years; >50 years
2. ER status: negative; positive
3. Tumour size: ≤50mm; >50mm
4. Clinical axillary node involvement:

yes; no

1. Inflammatory or locally advanced:

yes; no

Randomisation

**Arm A1**

**4 x EC followed by 4 x T**

EC: as for tAnGo

T: 175mg/m^2^ q2 weekly

**Arm A2**

**4 x T followed by 4 x EC**

T: 175mg/m^2^ q2 weekly

EC: as for tAnGo

**Arm B1**

**4 x EC followed by 4 x T+G**

EC: as for tAnGo

T: 175mg/m^2^ q2 weekly

G: 2000mg/m^2^ q2 weekly

**Arm B2**

**4 x T +G followed by 4 x EC**

T: 175mg/m^2^ q2 weekly

G: 2000mg/m^2^ q2 weekly

EC: as for tAnGo

SURGERY

***Abbreviations*** ER=estrogen receptor; HER2=human epidermal growth factor receptor 2; C=Cyclosphomide; E=Epirubicin; M=Methotrexate; F=5Fluorouracil; T=Paclitaxel; G=Gemcitabine
