# Supplementary material for: The Relationship between Common Genetic Markers of Breast Cancer Risk and Chemotherapy-Induced Toxicity: A Case-Control Study
Source: PLoS One. 2016 Jul 8;11(7):e0158984. doi: 10.1371/journal.pone.0158984 (PMC4938564; doi:10.1371/journal.pone.0158984)
Supplement: S1 Table — (DOCX) [file pone.0158984.s002.docx]

**S1 Table: Summary of clinical trials contributing to the PGSNPS study^1^**

|  | **NEAT (and BR9601)** | **tAnGo** | **NeotAnGo** |
| --- | --- | --- | --- |
| Ethics No. of Trial | LREC no. 96/285M | MREC no. 00/7/44 | COREC no. 04/MRE01/60 |
| Principle research question | In early breast cancer, is adjuvant chemotherapy with Epirubicin (E) following by Cyclophosphamide (C), Methotrexate and Flurouracil (CMF) significantly superior to CMF alone in terms of DFS and OS? | In early breast cancer, does adjuvant EC following by Paclitaxel and Gemcitabine (TG) improve disease-free survival (DFS) compared with EC-T alone? | What is the role of G in a sequential neoadjuvant chemotherapy regimen of EC and T and the role of sequencing of these treatment components in terms of outcome in high risk, invasive breast cancer? |
| Trial Status and No. of women recruited | Closed 07/01  Recruited 2401 | Closed 11/04  Recruited 3152 | Closed 09/07  Recruited 831 |
| Primary Endpoints | 5 year relapse free survival (RFS)  and overall survival (OS) | 5 year disease free survival (DFS) | Complete pathological response rates after neoadjuvant treatment. |
| Secondary Endpoints | 10 year RFS and OS; toxicity; Quality of Life (QoL); dose intensity | 5 & 10 year OS;  10 year DFS; toxicity; dose intensity:  Serious Adverse Drug Reactions. | Clinical and radiological response after 4 & 8 cycles; DFS and OS; QoL; prognostic & predictive markers analysis; Pathological response outcome measures. |

^1^Table copied with permission from Abraham et al (23)
