# Supplementary material for: The Relationship between Common Genetic Markers of Breast Cancer Risk and Chemotherapy-Induced Toxicity: A Case-Control Study
Source: PLoS One. 2016 Jul 8;11(7):e0158984. doi: 10.1371/journal.pone.0158984 (PMC4938564; doi:10.1371/journal.pone.0158984)
Supplement: S2 Table — (DOCX) [file pone.0158984.s003.docx]

**S2 Table – Patient Characteristics**

|  | **N (%)** | | | | |
| --- | --- | --- | --- | --- | --- |
|  | **NEAT (N=318)** | **tAnGo (N=876)** | **Neo-tAnGo (N=427)** | **E-CMF^a^ (N=56)** | **All PGSNPS (N=1677)** |
| **Age group** |  |  |  |  |  |
| ≤50 years | 178 (56.0) | 437 (49.9) | 268 (62.8) | 23 (41.0) | 906 (54.0) |
| >50 years | 140 (44.0) | 439 (50.1) | 159 (37.2) | 33 (59.0) | 771 (46.0) |
| **BMI** |  |  |  |  |  |
| <18.5 | 1 (0.3) | 7 (0.8) | 4 (1.0) | 5 (8.9) | 17 (1.0) |
| ≥18.5 and <25 | 141 (44.3) | 368 (42.0) | 175 (41.0) | 26 (46.4) | 710 (42.4) |
| ≥25 and <30 | 102 (32.1) | 304 (34.7) | 150 (35.1) | 13 (23.2) | 569 (33.9) |
| ≥30 | 62 (19.5) | 197 (22.5) | 97 (22.7) | 11 (19.7) | 367 (21.9) |
| missing | 12 (3.8) | 0 (0.0) | 1 (0.2) | 1 (1.8) | 14 (0.8) |
| **Performance status** |  |  |  |  |  |
| 0 | 201 (63.2) | 805 (91.9) | 383 (89.7) | 25 (44.6) | 1414 (84.3) |
| ≥1 | 98 (30.8) | 71 (8.1) | 18 (4.2) | 2 (3.6) | 189 (11.3) |
| missing | 19 (6.0) | 0 (0.0) | 26 (6.1) | 29 (51.8) | 74 (4.4) |
| **ER status** |  |  |  |  |  |
| negative | 107 (33.6) | 314 (35.8) | 136 (31.9) | 15 (26.8) | 572 (34.1) |
| positive | 191 (60.1) | 562 (64.2) | 291 (68.1) | 41 (73.2) | 1085 (64.7) |
| missing | 20 (6.3) | 0 (0.0) | 0 (0.0) | 0 (0.0) | 20 (1.2) |
| **HER2 status** |  |  |  |  |  |
| negative | 223 (70.1) | 555 (63.4) | 260 (60.9) | 23 (41.1) | 1061 (63.3) |
| positive | 45 (14.2) | 118 (13.4) | 103 (24.1) | 19 (33.9) | 285 (17.0) |
| missing | 50 (15.7) | 203 (23.2) | 64 (15.0) | 14 (25.0) | 331 (19.7) |
| **Tumour grade** |  |  |  |  |  |
| well | 9 (2.8) | 28 (3.2) | 9 (2.1) | 3 (5.4) | 49 (2.9) |
| moderate | 121 (38.1) | 287 (32.8) | 134 (31.4) | 19 (33.9) | 561 (33.5) |
| poor | 187 (58.8) | 559 (63.8) | 148 (34.7) | 34 (60.7) | 928 (55.3) |
| missing | 1 (0.3) | 2 (0.2) | 136 (31.8) | 0 (0.0) | 139 (8.3) |
| **Nodal status** |  |  |  |  |  |
| negative | 103 (32.4) | 197 (22.5) | 220 (51.5) | 25 (44.6) | 560 (33.3) |
| 1-3 positive | 163 (51.3) | 367 (41.9) | 0 (0.0) | 25 (44.6) | 555 (33.1) |
| 4+ positive | 51 (16.0) | 312 (35.6) | 0 (0.0) | 5 (9.0) | 368 (21.9) |
| missing | 1 (0.3) | 0 (0.0) | 207 (48.5) | 1 (1.8) | 194 (11.7) |
|  |  |  |  |  |  |
| *continued on next page* |  |  |  |  |  |
| **Randomised treatment regimen** |  |  |  |  |  |
| CMF | 156 (49.1) | - | - | - | 156 (9.3) |
| E-CMF | 162 (50.9) | - | - | 56 (100) | 218 (13.0) |
| EC-T | - | 451 (51.5) | 101 (23.7) | - | 552 (32.9) |
| EC-TG | - | 425 (48.5) | 106 (24.8) | - | 531 (31.7) |
| T-EC | - | - | 109 (25.5) | - | 109 (6.5) |
| TG-EC | - | - | 111 (26.0) | - | 111 (6.6) |

^a^Patients not taking part in a clinical trial recruited from Cambridge University Hospitals NHS Foundation trust

***Abbreviations*** ER=estrogen receptor; HER2=human epidermal growth factor receptor; E=Epirubicin, C=Cyclosphomide, M=methotrexate, F=5Fluorouracil, T=paclitaxel, G=gemcitabine
